# Predicted as observed? How to identify empirically adequate theoretical constructs

**DOI:** 10.3389/fpsyg.2022.980261

**Published:** 2022-12-01

**Authors:** Erich H. Witte, Adrian Stanciu, Frank Zenker

**Affiliations:** ^1^Institute for Psychology, University of Hamburg, Hamburg, Germany; ^2^Data and Research on Society, GESIS-Leibniz Institute for the Social Sciences, Mannheim, Germany; ^3^Department of Philosophy, Boğaziçi University, Istanbul, Turkey

**Keywords:** crowdsourcing hypothesis test, dissonance theory, empirical adequacy, Paul Meehl, meta-analysis, personality research, precognition, theory construction

## Abstract

The identification of an empirically adequate theoretical construct requires determining whether a theoretically predicted effect is sufficiently similar to an observed effect. To this end, we propose a simple similarity measure, describe its application in different research designs, and use computer simulations to estimate the necessary sample size for a given observed effect. As our main example, we apply this measure to recent meta-analytical research on precognition. Results suggest that the evidential basis is too weak for a predicted precognition effect of *d* = 0.20 to be considered empirically adequate. As additional examples, we apply this measure to object-level experimental data from dissonance theory and a recent crowdsourcing hypothesis test, as well as to meta-analytical data on the correlation of personality traits and life outcomes.

“*I am deliberately setting aside statistical significance testing*, *or the setting up of confidence intervals* […]”

(Meehl, 1990; p. 128)

## Introduction

As classical empirical findings fail to replicate and empirical studies often prove to be poorly conducted (Gervais, 2021; Nosek et al., 2022), the *replication crisis* or *confidence crisis* presents a major impasse for behavioral science (Fleck, 1935; Kuhn, 1962). While the motives for employing questionable research practices (Gelman and Carlin, 2014; Gelman, 2018) and the limitations of research methods (Kerr, 1998) are increasingly better understood, most reform proposals today recommend *transparency measures* (e.g., study pre-registration or registered replications; Fiedler and Prager, 2018; Klein et al., 2018). Less frequently addressed is that scientific progress requires good theoretical constructs (Meehl, 1978; Gigerenzer, 1998; Miłkowski et al., 2019; Muthukrishna and Henrich, 2019; Oberauer and Lewandowsky, 2019; Eronen and Romeijn, 2020; van Rooij and Baggio, 2020; Cornelissen et al., 2021; Eronen and Bringmann, 2021; Gervais, 2021; Irvine, 2021).

A good theoretical construct minimally allows for an empirically adequate prediction. A theoretical construct “is empirically adequate exactly if what it says about the observable things and events in the world is true—exactly if it “saves [or captures] the phenomena” (van Fraassen, 1980, p. 12). In the context of experimental research, this means that the effect that is predicted by a theoretical construct must be sufficiently *similar* to a relevant observed effect.

The development of an empirically adequate construct depends on high-quality observations. But even observations of the highest quality cannot automatically generate a theoretical construct that offers a *non-circular* justification for why a future event occurs as predicted.^1^ Because a theoretical construct must deductively entail its prediction *before* observations are made, a non-circular approach to predicting a phenomenon of interest thus requires a *deductive* approach to the development of empirical adequate theoretical constructs (Popper, 1959; Lakens, 2013; Lakens et al., 2018),

We begin by summarizing why the empirical adequacy of a theoretical construct should be evaluated *independently* of statistical elements (Meehl, 1990; p. 128) and review the shortcomings of extant evaluative approaches. To this end, we propose a new formal measure that is independent of statistical elements, thus enabling a *direct* comparison between theory and observation. The intended application for this measure is theory construction. To demonstrate its use value, we exemplarily evaluate recent meta-analytical findings on *precognition* (Bem et al., 2016). Additional examples, as well as a description of how this measure can be applied under various research designs, are provided in Supplementary Appendix S1, S2.

## Summary

Evaluating whether a theoretical prediction agrees with observations requires a theory-accommodating approach. But if this approach combinates theoretical and statistical aspects, then the evaluative outcome depends on the *variance* of error-prone observations. Consequently, one cannot be sufficiently certain about the accuracy of observations to which the theoretical prediction is compared. Since this uncertainty transfers to the evaluative outcome, the question of whether a theoretical prediction agrees with observations should be addressed *independently* of how observations vary (Meehl, 1990, 1992, 1997).

Yet the opposite holds if a standardized effect size measure such as Cohen’s *d* = (*m*_1_ – *m*_0_) / *s* is used to quantify the observations. This measure combines the observed *mean difference* (*m*_1_ – *m*_0_) with the statistical element of the observed *standard deviation* (*s*). A theoretical construct, however, predicts only (*m*_1_–*m*_0_), yet not *s*. This makes a standardized effect size measure an inappropriate formal tool to evaluate the empirical adequacy of a theoretical construct.

A theoretical construct contrasts most starkly with an inductive generalization that states a *directional* hypothesis. Because a directional hypothesis is informative only relative to its inductive basis, it can merely “predict” the pattern of past observations it subsumes. A theoretical construct, by contrast, is informative beyond this basis (see our note 1). Moreover, the construct must predict future observations not as a directional but as a *point*-specific effect. Otherwise, one simply cannot evaluate whether the theoretically predicted mean agrees with the observed mean.

## Shortcomings of the inductive strategy

### Standard deviation

The observed standard deviation (*s*) is a measure of the variance of observations. The observed variance depends on the extent to which an empirical setting is subject to uncontrolled (random) influences. Other things being equal, empirical settings that are more rigorously controlled for (random) influences go along with reduced observed variance, i.e., a smaller *s*. Compared to a less rigorously controlled setting, therefore, the value of Cohen’s *d*-measure increases.

Since the observed standard deviation quantifies the variance of error-prone observations, an observed effect must be related to a probability distribution. This process is known as *standardization*. Once standardized, the observed effect becomes a statistic of an entire sample of observations that can no longer be related *directly* to a theoretically predicted effect. With a standardized observed effect, therefore, one cannot evaluate the *similarity* between what a theoretical construct predicts and what a measurement instrument records. Instead, one evaluates the relative position of statistically transformed measurement scores on a measurement scale against a random distribution.

A statistical test relies on the observed standard deviation to evaluate whether the observed effect differs statistically significantly from a null hypothesis. A *t*-test, for instance, can often show that a *large* difference between the observed means in the experimental and the control group is statistically significant. But the standard deviation *combines* several causes that contribute to the observed variance (e.g., the sample selection process, the experimental implementation, the validity and the reliability of the in- and dependent variables, and the random influences on an empirical setting). Thus, a theoretically predicted and an observed effect may well agree. But if the observed effect depends on the observed standard deviation, then its statistical significance is an *insufficient* criterion to evaluate a theoretical construct as empirically adequate.

### Parameter estimation

Parameter estimation is an *inductive* strategy to separate systematic patterns from non-systematic noise in data. A parameter operates at the level of statistics rather than the level of measurement. ‘Parameter’ thus refers not to the properties of observations but those of data (e.g., their central tendency as measured by the mean, or the strength of associations between variables as measured by correlation or regression coefficients). Since data provide the basis for a parameter estimate, its accuracy is informed by statistical procedures that evaluate the parameter against the observed variance. The latter results from the variation of behavioral responses and measurement shortcomings. A given measurement instrument, therefore, captures both a relevant phenomenon *and* random influences (e.g., due to participants’ salient memories, chronic moods, or even the weather).

This leads to three complications in estimating a parameter accurately. First, since perfectly error-free observations are impossible, the accuracy of a parameter must be evaluated against the observed variance by using statistical procedures (that rely on a significance level *α* and an associated probability level *p*). Such procedures are often subjective and need not be reliable (see *p*-harking, Kerr, 1998; *p*-hacking, Simmons et al., 2013). Crucially, statistical procedures cannot distinguish whether the observed variance results from measurement shortcomings or rather from uncontrolled (random) influences on an empirical setting.

Second, what matters for scientific discovery is the size of the parameter estimate. For instance, a small observed mean difference between people’s political orientation that varies with color preferences presumably *fails* to be a substantially meaningful finding. Whereas a similarly small observed difference that varies with cultural background presumably would be substantially meaningful. This finding, however, should be further explored only if it is sufficiently large. But recent *meta*-meta-analyses (Olsson-Collentine et al., 2020; Schauer and Hedges, 2020; Linden and Hönekopp, 2021) strongly suggest that individual published studies across different behavioral science domains typically report observed object-level effects that are small and homogenous (read: small *d*, small *s*) or medium-to-large and heterogeneous (read: large(r) *d*, large *s*). A small observed variance thus tends to go along with a small observed mean effect. Whereas the findings of individual object-level studies that are sufficiently large to be further explored go along with a large observed variance. This necessarily results in a *vague* impression of the parameter that an empirical adequate theoretical construct would have to predict.

Third, a parameter estimate is useful for theory construction only if its inductive basis accurately captures an observed effect in a relevant population. Considerations of test-power and sample representativity dictate the use of sufficiently large samples to discover systematic behavioral patterns (law of large numbers). In small samples, by contrast, these patterns are likely truncated by uncontrolled (random) influences, resulting in inaccurate parameter estimates. Generally, large samples allow for more accurate parameter estimates if the underlying distribution of observations is uniform.

Among the widely used tools to estimate parameters are Cohen’s *d*-measure, confidence intervals, and tools that rely on inductive model fitting and probabilistic distributions.

#### Cohen’s *d-*measure

The goal of null-hypothesis significance testing is to determine whether an observed object-level effect differs significantly from a random effect. Relative to a predefined significance level *α* and an associated probability level *p*, the statistical significance of an observed effect indicates the probability of observing this effect under the null hypothesis. But this says nothing about whether the null or the alternative hypothesis is true or whether the observed object-level effect is relevant for theory construction. For theory construction, therefore, the statistical significance of an observed object-level effect is merely a necessary criterion. In addition, publications should also report the observed object-level effect’s size.

Among the available tools to calculate the observed effect size, standardized effect size measures are often preferred because they weigh the observed effect by the observed variance, thus providing a robustness check for the observed effect. As one of the most widely used measures in behavioral science (Schäfer and Schwarz, 2019), for instance, Cohen’s standardized *d*-measure *d* = (*m*_1_ – *m*_0_) / *s* (Cohen, 1977) weighs the observed mean difference (*m*_1_ – *m*_0_) between the experimental (*m*_1_) and the control group (*m*_0_) by the pooled standard deviation in both groups (*s*). It should be easy to see that, if (*m*_1_ – *m*_0_) is constant, then the *d*-value is sensitive to the observed variance captured by *s*.

Even if an experimental study that relies on the *d*-measure would report a very *large* statistically significant effect, this is insufficient to motivate the development of a theoretical construct for it. To be theorized, after all, is the *true* parameter, rather than its ratio to the observed variance. The main challenge thus is to tease apart the causes that contribute to the observed variance (see above). Standardized effect size measures, however, simply cannot meet this challenge, making them inappropriate tools for theory construction research. Therefore, an additional layer of scrutiny must address the confidence that an inductive parameter estimates the true parameter.

#### Confidence intervals

A true parameter can be estimated with perfect accuracy only in theory. In praxis, (random) influences or measurement instrument shortcomings render a perfectly accurate parameter estimate unlikely. One can nevertheless state the parameter’s *expected* accuracy using a *confidence interval* (CI), the width of which depends on the level of significance *α*. To determine the CI, one simultaneously considers the observed mean difference, the observed variance, the level of significance, and the sample size. This is formally given as CI=d±z×(s/√n).

Like Cohen’s *d*-measure, however, a CI cannot determine whether a true effect (e.g., the mean difference between two groups in a population) was estimated accurately because also a CI combines the mean difference with the statistical element *s*. Thus, the observed variance once again results in a *vague* impression of the parameter. Generally, unless the causes that contribute to the observed variance can be teased apart, vague observations will undermine theory construction research. And the one possible way of teasing these causes apart is to increase the sample size.

#### Inductive model fitting

Using inductive model fitting, researchers can address the complexity of human behavior by statistically modeling the associations between two or more estimated parameters, followed by testing the statistical model against a random model. Using various indexes (e.g., the Comparative Fit Index (CFI) or the Root Mean Square Error of Approximation (RMSEA)), a finite set of observations is compared against a class of statistical models (see the special issue on model selection, Myung et al., 2000; Burnham and Anderson, 2004). The model that best describes the data is said to be *identified* in the population (Bollen et al., 2010).

Inductive model fitting presupposes a reconstruction of the variance–covariance structure in the data. But fitting a statistical model to data inherits all attributes of the data (including errors due to measurement instrument shortcomings, uncontrolled random influences, non-uniform distributions, or outliers). So, although inductive model fitting improves over the estimation of a single parameter, its use-value for theory construction primarily depends on the quality of the data. Even the best-fitted model, however, cannot *unequivocally* tell meaningful data patterns from patterns owed to measurement instrument shortcomings or uncontrolled (random) influences. This holds regardless of whether the estimated parameter is statistically significant or whether the effect size is large. All an inductively fitted model can tell is whether data are described well.

Since model fitting is an *iterative* strategy, moreover, some parameters must be estimated before others, so that the associations between parameters can be specified to obtain a data-fitting model. The identification of the parameters that are to be estimated first would ideally rely on theoretical considerations. But when researchers fit a model to data, they instead often rely on *p*-harking or *p*-hacking strategies.

#### Bayesian probabilistic distributions

In the Bayesian approach to parameter estimation, the known probability of past observations is assumed to estimate the probability of (predicted) future observations. A theoretical construct can thus be evaluated based on the prior probability of a statistical model (Wagenmakers and Farell, 2004). The observed variance is here captured by the assumption that the theoretical construct is itself subject to variation. So, rather than evaluating the agreement between data and a single statistical model, Bayesians evaluate the agreement between data and a *distribution* of possible statistical models.

A theoretical construct is thus specified not as a single parameter, but as one that is embedded in a prior probability distribution (e.g., a normal or a Cauchy distribution). Of course, if this prior probability distribution accurately captures the true parameter, then a theoretical construct that is specified as a probability distribution may be useful for theory construction. What the *true* probability distribution is, however, one can never know. A Bayesian parameter estimate, therefore, depends not so much on the quality of the data, but more on a researcher’s (subjective) assumptions about the prior probability distribution (see Krefeld-Schwalb et al., 2018).

Since the theoretical construct is more likely to be associated with an upper and a lower probability bound than with a unique probability, the Bayesian approach to parameter estimation corresponds—except for the distribution of possible theoretical parameters—to the specification of a theoretical construct as an *interval* hypothesis (i.e., a two-point-hypothesis). Because the endpoints of this interval represent two distinct theoretical parameters, each endpoint must be *separately* evaluated against data. But the possibility of a separate evaluation of two theoretical parameters also shows that there is no genuine need to distribute them. After all, if the (subjective) *a priori* probabilities of both theoretical parameters are independent, then as one parameter is assigned probability 1, the other can be assigned probability 0.

## Toward a deductive strategy: Paul Meehl’s corroboration index

In the context of theory construction research, probably the first in behavioral science to recognize a problem in relating the theoretically predicted effect to the sample statistic *s* was Meehl (1990). Against the background of Lakatos’ (1978) “core vs. protective belt”-model of empirical theories—which recognizes that making suitable adjustments to the protective belt can (in principle forever) deflect the empirically inadequate predictions that constitute a theory’s *falsification instances* away from the core—Meehl argued that a formal measure for the empirical adequacy of a theoretical construct should *ignore s*.

“To construct a crude [corroboration-]index of a theory’s [predictive] track record, one first amends the earlier Popper to the later Popper by shifting emphasis from falsification to verisimilitude. […] Meanwhile, we require of a candidate index that it somehow reflect how bad a numerical “miss” the experimenter chalks up against [the theory] T. […] We are examining the relationship between T and its track record in predicting numerical values of [a hypothesis] H, *ignoring the stochastic slippage* between H and the data set that is the main concern of the statistician.”

(Meehl, 1990; p. 128)

Meehl’s corroboration index (*C_i_*) is the following:


(1) $$C_{i}=\left(Cl\right)\times \left(In\right)\;$$

where *Cl* = the closeness of observed data to the theoretical prediction;

*In* = the intolerance of the theory (e.g., the standardized precision of a prediction).

These terms can be expanded:


(2) Cl=1–(D/S)

where *D* = the deviation of observed data from the tolerance interval of the theory;

*S* = “Spielraum,” i.e., the expected range of observed data regardless of whether the theory is true; and


(3) In=1–(I/S)

where *I* = the interval tolerated by the theory (or the raw precision of a theoretical prediction).

For a given experiment, the index *C_i_* is the product of the *closeness* of the data to the theoretical prediction (*Cl*) and the *intolerance* of a theory (*In*). Thus, large values of *C_i_* are expected for an empirically adequate theoretical construct and small values of *C_i_* for an empirically inadequate one. Although several critics considered the *C_i_* measure overly complex (see the special issue of *Psychological Inquiry*, including Meehl 1990), Meehl (1992) rightly replied that formal measures are needed to develop empirically adequate theoretical constructs. Yet, Meehl’s key insight—that a formal measure to evaluate the empirical adequacy of a theoretical construct should *ignore* the statistical element *s*—further awaits uptake. Researchers instead continue to rely on statistical considerations (e.g., CIs, *t*, *d*, etc.) or on model-fitting approaches that combine theoretical with statistical elements.

Heeding Meehl’s insight, we propose the *similarity index I*_SIM_ as an alternative formal measure, one far simpler than *C_i_*.

## The similarity index

As we saw, if a parameter is induced from an interval of observations, then the parameter captures the uncontrolled (random) influences on an empirical setting that are represented by *s*. Although this parameter may (misleadingly) be referred to as a theoretical construct, this construct is as *vague* as the underlying interval of observations is wide. An inductive parameter, therefore, is at most as informative as a two-point, directional alternative hypothesis (H_1_). But a directional alternative hypothesis cannot stand in the *one-to-one* relation between prediction and observation that is required to evaluate whether a theoretical construct is empirically adequate (Klein, 2014; Szucs and Ioannidis, 2017; Gelman, 2018). Only a point-specific theoretical construct can do so.

For this reason, Meehl (1990) argued that the evaluation of the empirical adequacy of a theoretical construct should ignore *s*. Once the evaluation is independent of *s*, it pertains only to the *similarity* between a predicted and an observed mean difference in a sample. This is precisely what the similarity index *I*_SIM_ captures (see 4).


(4) $$I_{\mathrm{SIM}}=\frac{\left|m_{\mathrm{THEO}}-m_{0}\right|}{\left|m_{1}-m_{0}\right|}={\mathrm{ES}}_{\mathrm{THEO}}/{\mathrm{ES}}_{\mathrm{OBS}}$$

ES, effect size.

*m*_THEO_, the theoretically predicted mean.

*m*_1_, the observed mean in the treatment group.

*m*_0_, the observed mean in the control group.

*m*_THEO_ – *m*_0_, the theoretically predicted mean difference (ES_THEO_).

*m*_1_ – *m*_0_, the empirically observed mean difference (ES_OBS_).

A formal measure for the empirical adequacy of a theoretical construct should satisfy several criteria that are relevant to theory construction. First, an experimentally observed phenomenon must be independent of the measurement scale that a given measurement instrument presupposes. Second, any two phenomena that are recorded on distinct measurement scales must remain comparable. Third, observations must remain stable under theoretically plausible transformations.

But if different measurement scales are made comparable by a transformation into *z*-values, then recourse to the inductive element *s* entails that the measurement quality of the empirical setting is retained. A *z*-transformation thus *inherits* information originating from the uncontrolled (random) influences on an empirical setting. This is problematic for theory construction research because, given that *s* as a property of observations *lacks* a theoretical counterpart, recourse to *s* “blurs” the evaluation of the empirical adequacy of a theoretical construct.

*I*_SIM_ uses a transformation that avoids *s*. The comparability of observations that are recorded on different measurement scales is guaranteed because a ratio of differences is invariant under the addition of a constant or multiplication by some factor.^2^ *I*_SIM_ also guarantees that the direction of the observed effect can be interpreted. This matters for evaluating whether the observed effect leans toward the experimental or the control group. If the direction of the observed effect and the theoretically predicted effect agree, then *I*_SIM_ is invariant concerning the order of means. That the same mathematical signs (+, −) now appear in the numerator and the denominator of *I*_SIM_ can be neglected. Whereas if the direction of the observed effect and that of the theoretically predicted effect differ, then distinct mathematical signs indicate that the prediction fails to agree with observations. In this case, *I*_SIM_ is set to 0.

Using *I*_SIM_, the theoretically predicted effect can thus be compared *directly* to the observed effect. A direct comparison should arguably also apply if a theoretically predicted effect is compared to a meta-analytically estimated population effect that is aggregated from the results of independent replication studies. But the opposite is the case if this comparison relies on a standardized effect size measure such as Cohen’s *d*, which is widely used for this purpose today. Sometimes, indeed, the observed *d*-value simply stands in for the estimated population effect.

The intended application for *I*_SIM_ is a rigorously controlled empirical setting where participants are randomly allocated to the experimental and the control group, respectively are randomly selected as study participants in a correlational study.^3^ Since the use of this kind of setting to evaluate the empirical adequacy of a *directional* H_1_ undermines all efforts at controlling the setting, a rigorously controlled empirical setting should exclusively serve to evaluate the high-risk prediction that only a point-specific theoretical construct can offer.

### The similarity between theory and observations

The agreement between a theoretical prediction and observations is *perfect* if the ratio between both is one, i.e., ES_THEO_ / ES_OBS_ = 1.00. A perfectly empirically adequate prediction, however, is a strong idealization because even the most rigorously controlled empirical setting is subject to some uncontrolled (random) influences and errors. So, even if a theoretical construct predicts a population effect perfectly (i.e., ES_THEO_ = ES_POP_), a measurement instrument with imperfect reliability or random influences on an empirical setting do entail that the observed effect will be “blurred.” A formal measure for the empirical adequacy of a theoretical construct, therefore, can only *approximate* the agreement between a theoretical prediction and observations.

Analytically, the agreement between a theoretical prediction and observations varies between a *match* (*I*_SIM_ = 1.00) and a *mismatch* in one of two directions (*I*_SIM_ = 0 and *I*_SIM_ > > 1). The reason for a mismatch—namely whether the theoretical construct predicts an empirically inadequate effect or whether the observed effect is subject to random influences—can be teased out by collecting additional data, i.e., by increasing the sample size *n*. If the values of *I*_SIM_ cluster around 1 as *n* increases, this indicates that the theoretically predicted effect approximately matches a relevant population effect (law of large numbers). As the observed effect thus progressively converges onto the population effect (ES_OBS_ = ES_POP_), it can eventually be excluded that random influences account for the observations. Thus, one gains evidence that the theoretically predicted effect is *empirically adequate*. This case is perfect for theory construction because the theoretical construct can be adopted into a theory.

Whereas if values of *I*_SIM_ never cluster around 1 as *n* increases, then the theoretical prediction is *empirically inadequate*. This means one gains evidence that the theoretically predicted effect misrepresents the population effect, wherefore the theoretical construct requires adjustment. Subsequently, a new theoretically predicted effect must be separately evaluated using new observations.

### The similarity interval

Defining the range of acceptable deviations from a perfect match requires an interval of the form [*x* < *I*_SIM_ = 1.00 < *y*]. The purpose of this *similarity interval* (SI) is distinct from that of a *confidence interval* (CI). When a population effect (ES_POP_) is estimated from observations, a CI handles randomly distributed “noise” in an empirical setting by stating the interval within which ES_POP_ is expected to lie to some predefined probability (see the section *Parameter Estimation*). The SI, by contrast, differentiates between evidence for and against the empirical adequacy of a theoretical construct by stating the probability that the theoretically predicted effect is similar to observations if a study is *repeated* numerous times.

The SI is motivated by two constraints. First, an empirically adequate theoretical construct must neither grossly under- nor grossly over-predict the population effect (ES_THEO_ ≅ ES_POP_). Second, provided the first constraint holds, if the theoretically predicted effect keeps approximating the observed effect as the number of study repetitions increases, then the theoretically predicted effect becomes increasingly more promising as a parameter for theory construction because the prediction remains empirically adequate.

The SI particularly facilitates the identification of a *preliminary* match between a theoretically predicted effect (ES_THEO_) and an observed effect (ES_OBS_), because an *I*_SIM_-based evaluation is fallible—future studies may lead to an opposite evaluation. We define a preliminary match using an SI with bounds of [0.80;1.20]. If the *I*_SIM_ value lies within these bounds, then the theoretical prediction is preliminarily empirically adequate. The bounds [0.80;1.20] are informed by 10,000 simulated study repetitions (see the section *Simulated Data and Results*). For instance, given *n*_0_ = *n*_1_ = 1,000 participants, our simulations show that if the population effect is a *medium* effect, ES_POP_ = 0.50, then *I*_SIM_-values fall within this SI in approximately 99% of 10,000 study repetitions. And, given *n*_0_ = *n*_1_ = 100 participants in each study condition, if the population effect is a *large* effect, ES_POP_ = 1.00, then *I*_SIM_-values fall within the SI in approximately 95% of 10,000 repetitions.

Since a *small* sample suffices to detect a *large* population effect under small error-rates, whereas detecting a *small* population effect requires a *large* sample, the application of a 99%-SI to the small to medium effects that are normally observed in behavioral science would require unrealistically large samples (Linden and Hönekopp, 2021). Given the conventional error rate of 5%, however, already a 95%-SI can suffice as an evidence-based criterion to decide whether a theoretical construct can be accepted as empirically adequate, whether it should be improved, or whether additional data should be collected.

### Simulated data and results

If simulations approximate the universe of possible observed effects, they are useful to explore the stability of effects that real studies would observe (see Morris et al., 2019). Real observations are made in samples drawn from some population of interest. But researchers typically cannot access the entire population, neither in real life nor in simulations. To account for the ultimately unknown observed variance, real observations are treated statistically as a *t*-distribution, which is sensitive to *n*. As *n* increases, a *t*-distribution approximates the normal distribution that is expected for a population (central limit theorem).

We therefore simulated data from *t*-distributions in a universe of study settings that comprises 10,000 repeated individual studies of the same effect. A study setting is characterized by the means observed in the control (*m*_0_) and the experimental group (*m*_1_) and by the sample size (*n*_0_ = *n*_1_). All simulations were conducted in R (R Core Team, 2021) using the packages tidyverse (Wickham et al., 2019), dplyr (Wickham et al., 2021), and effsize (Torchiano, 2020).

**Figure 1:**
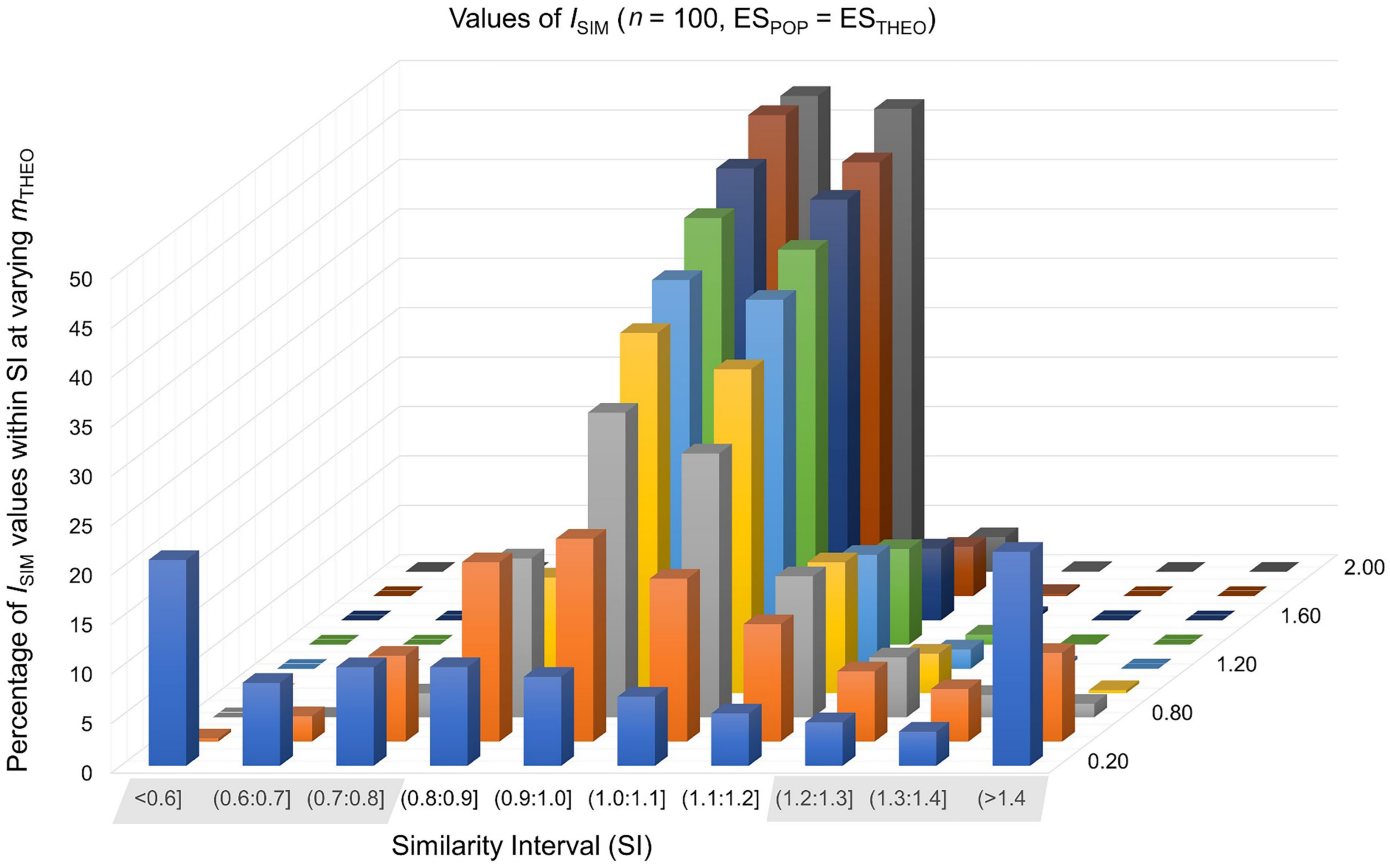
Values of *I*_SIM_ were calculated in 10,000 simulated study-settings with *n*_0_ = *n*_1_ = 100 under the assumption that the theoretically predicted effect matches the population effect. Each row of this graph represents different values of *m*_THEO_.

In the first of two basic scenarios, where the theoretically predicted effect *matches* the population effect (ES_THEO_ = ES_POP_), the sample size of a study setting was *n*_0_ = *n*_1_ = 20, 30, 50, 100, 300, or 1,000. In the control group the observed mean was null (*m*_0_ = 0) and in the experimental group *m*_1_ = 0.20, 0.50, 0.80, 1.00, 1.20, 1.40, 1.60, 1.80, or 2.00. In this way, we simulated 54 study settings times 10,000 repetitions, calculating the similarity index *I*_SIM_ separately for each repetition of a study setting (see formula 4). For the percentages of *I*_SIM_-values falling inside and outside the similarity interval SI, see Table 1 and Figure 1.

**Table 1:** True predictions [ES_THEO_ = ES_POP_ equals (*m*_THEO_ – *m*_0_) = (*m*_POP_ – *m*_0_)]: expected *I*_SIM_-values for varying values of *m*_THEO_ and *n*.

*m*_THEO_ = *m*_POP_	*n*	<0.6]	(0.6,0.7]	(0.7,0.8]	(0.8,0.9]	(0.9,1.0]	(1.0,1.1]	(1.1,1.2]	(1.2,1.3]	(1.3,1.4]	>(1.4
0.20											
	20	52.2	5.55	5.67	5.34	4.52	3.44	2.69	2.3	1.96	16.33
	30	45.89	6.64	6.43	5.7	4.84	4.14	3.61	2.78	1.92	18.05
	50	35.97	7.86	7.43	7.29	6.29	5.06	4.24	3.49	2.75	19.62
	100	20.84	8.4	9.97	9.98	9	7	5.3	4.39	3.46	21.66
	300	3.26	5.69	11.86	15.24	15.25	12.31	9.1	6.6	4.6	16.09
	1,000	0.04	0.71	5.62	17.65	25.46	21.84	13.84	6.94	3.87	4.03
0.50											
	20	18.04	8.61	10.18	10.67	8.86	7.93	6.07	4.77	3.71	21.16
	30	10.25	8.03	10.77	12.29	11.78	9.09	7.05	5.74	4.36	20.64
	50	3.32	6.03	10.57	14.8	15.65	12.71	9.16	6.33	4.28	17.15
	100	0.33	2.58	8.69	18.15	20.52	16.48	11.87	7.1	5.31	8.97
	300	0	0.04	1.96	15.78	32.69	28.31	13.69	5.02	1.51	1
	1,000	0	0	0	3.68	46.41	42.07	7.32	0.5	0.02	0
0.80											
	20	3.82	5.49	11.44	15.23	14.87	12.24	8.93	7.06	4.26	16.66
	30	1.1	4.02	10.5	16.51	18.03	15.48	10.74	7.25	4.96	11.41
	50	0.08	1.26	7.74	17.78	23.74	19.29	12.41	7.53	3.96	6.21
	100	0	0.05	2.42	16.08	30.82	26.69	14.27	6.06	2.24	1.37
	300	0	0	0.11	6.03	44.34	39.03	9.41	1.02	0.05	0.01
	1,000	0	0	0	0.28	49.5	49.18	1.04	0	0	0
1.00											
	20	1.19	3.96	10.87	16.73	17.35	15.2	10.68	6.71	4.87	12.44
	30	0.19	1.65	8.32	18.17	22.51	17.97	12.35	7.55	4.49	6.8
	50	0.01	0.29	4.33	17.45	28.02	22.9	13.84	7.24	3.09	2.83
	100	0	0	0.8	11.7	36.43	32.75	13.23	3.98	0.82	0.29
	300	0	0	0	2.75	48.25	43.03	5.77	0.2	0	0
	1,000	0	0	0	0.04	49.99	49.84	0.13	0	0	0
1.20											
	20	0.17	2.07	8.31	18.05	21.51	17.93	12.61	7.18	4.25	7.92
	30	0.03	0.57	5.43	18.69	25.74	21.59	13.15	7.29	3.51	4
	50	0	0.02	2.03	15.88	31.69	27.75	14.34	5.33	1.87	1.09
	100	0	0	0.25	9.25	39.33	37.34	11.53	1.95	0.33	0.02
	300	0	0	0	1.15	48.05	47.62	3.12	0.06	0	0
	1,000	0	0	0	0	49.58	50.4	0.02	0	0	0
1.40											
	20	0.02	1.03	6.74	17.44	24.80	20.73	13.02	7.28	4.04	4.90
	30	0	0.21	3.40	17.22	28.40	25.32	13.83	6.60	2.90	2.12
	50	0	0.01	0.95	13.18	36.19	30.6	13.48	4.04	1.15	0.4
	100	0	0	0.01	6.21	43.14	39.94	9.67	0.99	0.03	0.01
	300	0	0	0	0.46	49.71	48.36	1.47	0	0	0
	1,000	0	0	0	0	49.94	50.06	0	0	0	0
1.60											
	20	0	0.39	4.62	17.25	27.52	23.28	13.94	6.78	3.32	2.90
	30	0	0.08	2.02	15.50	33.03	27.58	13.67	5.17	1.96	0.99
	50	0	0	0.44	10.87	38.88	34.33	12.22	2.79	0.38	0.09
	100	0	0	0	4.06	45.70	42.55	7.25	0.43	0.01	0
	300	0	0	0	0.1	50.43	48.8	0.67	0	0	0
	1,000	0	0	0	0	50.4	49.6	0	0	0	0
1.80											
	20	0	0.19	2.83	16.56	30.69	25.19	14.10	6.30	2.43	1.71
	30	0	0.01	0.94	13.79	35.09	30.50	13.77	4.43	1.06	0.41
	50	0	0	0.1	8.07	42.21	36.61	10.85	1.83	0.28	0.05
	100	0	0	0	2.26	48.66	43.88	4.98	0.22	0	0
	300	0	0	0	0.03	49.82	49.99	0.16	0	0	0
	1,000	0	0	0	0	49.72	50.28	0	0	0	0
2.00											
	20	0	0.11	2.01	15.78	32.42	27.64	14.13	5.23	1.82	0.86
	30	0	0	0.49	11.42	38.40	32.96	12.47	3.33	0.71	0.22
	50	0	0	0.07	6.51	44.16	39.04	9.13	1	0.08	0.01
	100	0	0	0	1.45	48.14	46.84	3.49	0.08	0	0
	300	0	0	0	0	50.68	49.21	0.11	0	0	0
	1,000	0	0	0	0	50.62	49.38	0	0	0	0

Cells state the percentages of I_SIM_-values falling within specific I_SIM_ intervals for various sample sizes (n), based on 10,000 simulations per row; (, value not included; ], value included.

Findings are consistent with the claim that an empirical adequate theoretical construct is associated with values of *I*_SIM_ that fall inside the SI [0.80;1.20]. For example, values of *I*_SIM_ fall inside this SI in approximately 95% of study repetitions if the sample size is *n*_0_ = *n*_1_ = 100 and if (*m*_1_ – *m*_0_) = 1.00. In contrast, values of *I*_SIM_ fall inside this SI in approximately 67% of study repetitions given the same sample size and a smaller effect of (*m*_1_ – *m*_0_) = 0.50. This suggests that *n*_0_ = *n*_1_ = 100 suffices to evaluate a *large* theoretically predicted effect as preliminarily empirically adequate, whereas evaluating a *small* or *medium* theoretically predicted effect requires a considerably larger sample.

The second scenario, where the theoretically predicted effect *failed* to match the population effect (ES_THEO_ ≠ ES_POP_), examined how false positive and false negative predictions fare in our simulated universe of study repetitions. A false positive prediction occurs if the theoretically predicted effect is mistakenly identified as matching the population effect. And a false negative prediction occurs if the value of *I*_SIM_ falls outside the SI despite the theoretically predicted effect matching the population effect. In this scenario, we simulated four study settings where the theoretically predicted effect varied from small to large, and the population effect was either over- or underestimated. Notice that the relevant quantity to guide the identification of an empirically adequate theoretical construct here is not the *absolute* probability of detecting an empirically (in-)adequate prediction, but the *difference* between the probabilities of detecting one or the other kind of prediction.

**Figure 2:**
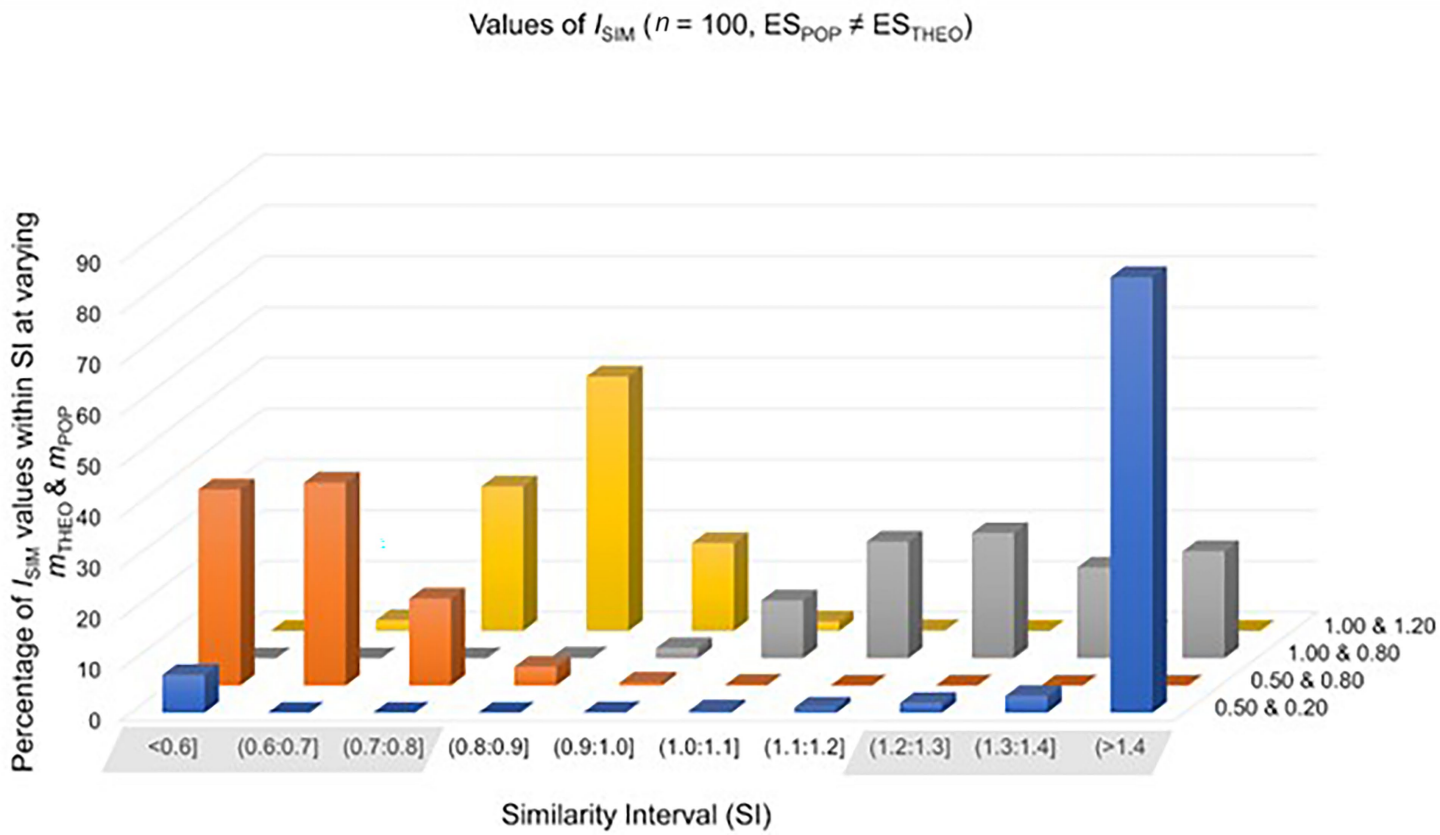
Values of *I*_SIM_ were calculated in 10,000 simulated study-settings with *n*_0_ = *n*_1_ = 100 under the assumption that the theoretically predicted effect does not match the population effect. Each row of this graph represents different combinations of *m*_THEO_ and *m*_POP_.

In each of the four study settings, the sample size was *n*_0_ = *n*_1_ = 20, 30, 50, 100, 300 or 1,000. In two of the four study settings, the theoretically predicted effect *overestimates* the population effect. Setting 1 simulated data from *t*-distributions representing a population effect of ES_POP_ = 0.20, whereas the theoretically predicted effect was ES_THEO_ = 0.50. Setting 2 simulated data from *t*-distributions representing a population effect of ES_POP_ = 0.80, whereas the theoretically predicted effect was ES_THEO_ = 1.00. In the remaining two study settings, the theoretically predicted effect *underestimates* the population effect. Setting 3 simulated data from *t*-distributions representing a population effect of ES_POP_ = 0.80, whereas the theoretically predicted effect was ES_THEO_ = 0.20. Setting 4 simulated data from *t*-distributions representing a population effect of ES_POP_ = 1.20, whereas the theoretical effect was ES_THEO_ = 1.00. All four study settings were repeated 10,000 times. For the percentages of *I*_SIM_-values falling inside and outside the SI, see Table 2 and Figure 2.

**Table 2:** False predictions (ES_THEO_ ≠ ES_POP_): expected *I*_SIM_-values given discrepancies between ES_THEO_ (*m*_THEO_ – *m*_0_) and ES_POP_ (*m*_POP_ – *m*_0_) for varying *n*.

*m*_THEO_ ≠ *m*_POP_	*n*	<0.6]	(0.6,0.7]	(0.7,0.8]	(0.8,0.9]	(0.9,1.0]	(1.0,1.1]	(1.1,1.2]	(1.2,1.3]	(1.3,1.4]	>(1.4
0.50 ≠ 0.20											
	20	28.08	0.84	1.47	2.70	3.38	3.86	4.18	3.98	3.56	47.95
	30	22.41	0.40	0.94	1.37	2.53	3.09	3.95	4.19	3.88	57.24
	50	16.11	0.03	0.11	0.54	1.19	2.21	2.99	3.86	4.15	68.81
	100	7.47	0	0	0.01	0.13	0.45	1.04	1.93	3.29	85.68
	300	0.59	0	0	0	0	0	0	0.09	0.39	98.93
	1,000	0	0	0	0	0	0	0	0	0	100
0.50 ≠ 0.80											
	20	45.21	18.25	13.53	8.23	5.09	3.08	1.66	1.15	0.76	3.04
	30	44.90	21.76	15.61	8.48	4.16	2.19	0.78	0.67	0.33	1.12
	50	42.86	28.41	17.45	6.74	2.66	1.1	0.43	0.17	0.07	0.11
	100	38.55	39.89	17.09	3.7	0.6	0.13	0.01	0.02	0.01	0
	300	31.46	59.73	8.68	0.13	0	0	0	0	0	0
	1,000	19.03	80.32	0.65	0	0	0	0	0	0	0
1.00 ≠ 0.80											
	20	0.80	0.69	2.53	5.76	10.07	12.96	12.61	10.64	8.69	35.25
	30	0.14	0.16	0.97	4.04	9.52	13.54	14.83	13.22	11.21	32.37
	50	0.03	0	0.09	1.51	6.88	14.29	18.59	17.29	13.49	27.83
	100	0	0	0	0.11	1.99	11.39	22.93	24.65	17.87	21.06
	300	0	0	0	0	0.07	2.97	25.10	42.35	22.06	7.45
	1,000	0	0	0	0	0	0.03	14.63	68.47	16.37	0.50
1.00 ≠ 1.20											
	20	4.72	13.52	23.39	23.79	15.41	8.48	4.53	2.37	1.66	2.13
	30	1.99	11.73	27.04	27.82	17.23	8.00	3.36	1.59	0.64	0.60
	50	0.26	7.22	29.28	36.03	19.12	6.02	1.53	0.37	0.09	0.08
	100	0	2.13	28.44	50.06	17.3	1.87	0.19	0	0.01	0
	300	0	0.02	19.69	73.84	6.41	0.04	0	0	0	0
	1,000	0	0	5.93	93.82	0.25	0	0	0	0	0

Cells state the percentages of *I*_SIM_-values falling within specific *I*_SIM_ intervals for various sample sizes *(n)*, based on 10,000 simulations per row; (, value not included; ], value included; *m*_POP_, the true value in the population (i.e., the numerator of *I*_SIM_); *m*_THEO_, the theoretical value in the population (i.e., the denominator of *I*_SIM_).

We first turn to cases where the theoretically predicted effect *overestimates* the population effect. Given a sample size of *n*_0_ = *n*_1_ = 100, values of *I*_SIM_ fall inside the SI in approximately 2% of repetitions of setting 1 (ES_POP_ = 0.20, ES_THEO_ = 0.50), compared to approximately 31% of repetitions of a study setting where the theoretically predicted effect matches the population effect (ES_THEO_ = ES_POP_ = 0.20). The 29% difference between false positive and true positives predictions increases as *n* increases (see Tables 1, 2). For the 2% of false positive predictions, the decision is clear: the theoretical construct requires adjustment. Whereas in case of the 31% true positive predictions, the identification of an empirically adequate construct would benefit from increasing *n*.

Further, given a sample size of *n*_0_ = *n*_1_ = 100, values of *I*_SIM_ fall inside the SI in approximately 36% of repetitions of setting 2 (ES_POP_ = 0.80, ES_THEO_ = 1.00), compared to approximately 88% of repetitions of a study setting where the theoretically predicted effect matches the population effect (ES_THEO_ = ES_POP_ = 0.80). The 53% difference between false positive and true positive predictions increases as *n* increases. In both cases, however, the decision to adjust the theoretical construct requires considerably larger samples to clearly distinguish a true positive from a false positive prediction.

We now turn to cases where the theoretically predicted effect *underestimates* the population effect. Given a sample size of *n*_0_ = *n*_1_ = 100, values of *I*_SIM_ fall inside the SI in approximately 4% of repetitions of setting 1 (ES_POP_ = 0.80, ES_THEO_ = 0.20), compared to approximately 88% of repetitions of a study setting where the theoretically predicted effect matches the population effect (ES_THEO_ = ES_POP_ = 0.80). The 84% difference between true positives and false positive predictions arguably suffices to evaluate the theoretical construct as empirically inadequate.

Finally, given a sample size of *n*_0_ = *n*_1_ = 100, values of *I*_SIM_ fall inside the SI in approximately 69% of repetitions of setting 1 (ES_POP_ = 1.20, ES_THEO_ = 1.00), compared to approximately 97% of repetitions of a study setting where the theoretically predicted effect matches the population effect (ES_THEO_ = ES_POP_ = 1.20). The 28% difference between false positives and true positive predictions suggests that it is more likely that values of *I*_SIM_ fall inside the SI if the theoretical prediction matches the population effect than otherwise.

We proceed to exemplify the application of *I*_SIM_ with a case study. Additional examples are provided in Supplementary Appendix S2.

### Case study: The psi-effect

The question of whether humans can cognize the future (aka *precognition* or *psi-*effect) has interested several scholars in psychology. The authors of the largest meta-analysis on the psi-effect to date (Bem et al., 2016), comprising 90 experimental studies of which 51 are peer-reviewed (see Bem et al., 2016; Supplementary Table S1), claim to have obtained decisive evidence *for* a psi-effect. Whereas some concluded from this that the psi-effect is real (e.g., Cardena, 2018), others argued that Bem et al.’s (2016) meta-analytical data leave it too unlikely that the psi-effect is real (e.g., Witte and Zenker, 2017).

Across the 51 peer-reviewed object-level psi-studies, the observed effect ranges from *d* = 0.02 to *d* = 0.21 (Bem et al., 2016). These two values describe a ratio of 1 : 9.7, indicating that the observed object-level effects are very heterogeneous. The heterogeneity of the observed object-level effects may suggest that the *average* psi-effect should be evaluated by combining a statistical inference strategy with an error account (Lord and Novick, 1968). This evaluation, however, would remain sensitive to how *n* and *s* vary across individual studies. But as statistical parameters, *n* and *s* lack theoretical meaning. Particularly *s* is merely a normalization factor to render several object-level effects comparable.

To achieve an evaluation that is independent of how *n* and *s* vary across the object-level studies, one should rather compare the point-specific ES_THEO_ *directly* to the point-specific ES_OBS_ in each study, *without* averaging the effect. To this end, Bem et al.’s (2016; Supplementary Table A1) meta-analytical findings can be re-analyze as follows:

1. As Bem himself proposed (Bem, 2011, p. 409, note 1), the theoretical psi-effect is specified as *d*_THEO_ = 0.20 using a scale of *z*-values where *s* = 1. Consequently, *d*_THEO_ = ES_THEO_. (A theoretical construct cannot reasonably predict a smaller psi-effect because it would be overlain by the standard measurement error.)
2. To control for the quality of the object-level studies, we exclude the 49 non-peer-reviewed object-level studies, retaining the 51 peer-reviewed ones (see Bem et al., 2016, Supplementary Table S1).
3. To eliminate the variation of *s*, the mean difference (*m*_1_ − *m*_0_) is calculated by multiplying the instance of ES_OBS_ in each peer-reviewed object-level study with that study’s observed *s*. This yields ES_OBS_ = (*m*_1_ − *m*_0_) / *s*, where *s* = 1.
4. For each peer-reviewed object-level study, *I*_SIM_ is computed as follows: (a) *I*_SIM_ = 0 if the mean difference is negative; (b) *I*_SIM_ is undefined if the between-group ES_OBS_-difference (treatment vs. control) is 0; otherwise, since *s* = 1, (c) *I*_SIM_ = (ES_THEO_ = 0.20 × *s*) / (ES_OBS_ × *s*) = (0.20 / ES_OBS_).

Because *s* has been eliminated, the 95%-SI [0.80;1.20] can be applied to each peer-reviewed object-level study individually. The two relevant parameters are ES_THEO_ = *d*_THEO_ = 0.20 relative to the sample size of an object-level study, and the percentage of ES_OBS_-instances that fall inside the 95%-SI given ES_THEO_ = *d*_THEO_ = 0.20.

The application of *I*_SIM_ indicates that, although each of the 51 peer-reviewed object-level studies was published as evidence *for* a psi-effect (Bem et al., 2016), the mean difference is negative (*I*_SIM_ = 0) in 16 studies (31% of 51 studies), that two studies show no difference (*I*_SIM_ is undefined), and that the *I*_SIM_-value falls outside the 95%-SI in 22 studies (43%). This means that ES_OBS_ is *insufficiently similar* to ES_THEO_.

In the remaining 11 studies (22% of 51 studies), where ES_OBS_ is *sufficiently similar* to ES_THEO_, the percentages of *I*_SIM_-values falling inside the 95%-SI (see Table 1) are nevertheless quite low: 33% (*n*_0_ = *n*_1_ = 100); 37% (150); 33% (99); 33% (100); 33% (100); 34% (109); 23% (49); 33% (100); 34% (111); 42% (201); 23% (50). This means that each study’s sample is *too small* to generate the evidence required to consider empirically adequate a theoretical construct that predicts ES_THEO_ = *d*_THEO_ = 0.20.

To appreciate the sample size that is needed to consider as empirically adequate a theoretical construct that predicts ES_THEO_ = *d*_THEO_ = 0.20, a one-sided *t*-test under *α* = 0.05 and test-power of (1 − *β*) = 0.80 already requires *n*_0_ = *n*_1_ = 101. Under *α* = *β* = 0.05, it even requires *n*_0_ = *n*_1_ = 201. The reason for the large samples is that the theoretically predicted effect is small enough to be accounted for exclusively by random influences on the empirical setting. But random influences are independent of ES_THEO_ and so lack theoretical meaning. Indeed, this is the reason why ES_THEO_ = *d*_THEO_ = 0.20 requires a statistical corroboration against random influences in the first place.

In sum, although ES_OBS_ is sufficiently similar to ES_THEO_ in 11 out of 51 peer-reviewed object-level studies, these 11 studies *individually* fail to provide the evidence required to consider as empirically adequate a theoretical construct that predicts ES_THEO_ = *d*_THEO_ = 0.20. Arguably, therefore, if the empirical adequacy of the theoretically predicted psi-effect had been evaluated before conducting additional studies, some research effort concerning the psi-effect could have been avoided.

## Discussion

Whether a theoretical construct adequately predicts future observations is a distinct question from whether a data-based parameter estimate (induced from past observations) deviates statistically significantly from a random distribution. This difference matters because behavioral science research regularly uses a data-based parameter estimate and its associated confidence bounds as a proxy for a theoretical construct. But a parameter that is estimated using a *z*-standardized effect size measure such as Cohen’s *d* cannot distinguish whether particularly a *small* observed *d*-value points to a mean difference that is too small to be observable, or rather to a large *s*. Without *making* this distinction, however, the evaluation of the empirical adequacy of a theoretical construct is out of reach.

The *I*_SIM_ measure and the SI fare better. Both together can inform the evaluation of the empirical adequacy of a theoretical construct because, if the inductive element *s* that serves to *z*-standardize measurements is avoided, then the observed mean difference *ceases* to be “blurred” by random influences. As this enables a *direct* comparison between the theoretically predicted and the observed mean-difference, the evaluation of the empirical adequacy of a theoretical construct is placed within reach. On how *I*_SIM_ and the SI can be applied beyond a simple experimental setting, see Supplementary Appendix S1. For additional examples, see Supplementary Appendix S2. To apply *I*_SIM_ and the SI to extant data, we provide an online tool at https://adrian-stanciu.shinyapps.io/Similarity-Index/.

### Practical implications

As behavioral science has come under scrutiny, *replication crisis* denotes that few previously “established” findings are independently replicable and that questionable research practices are regularly employed (e.g., Kerr, 1998; Klein, 2014; Irvine, 2021; Nosek et al., 2022). A familiar response to the replication crisis is to recommend measures that improve the quality of data (e.g., study pre-registrations, multi-lab projects, or open access to materials). Such measures constitute important elements of an inductive approach to parameter estimation. But some effort must also go toward developing theoretical constructs that logically entail an empirically adequate prediction, i.e., toward a deductive approach to theory construction.

A central limitation of the inductive approach to parameter estimation is exemplified by meta-analytical research. To arrive at robust meta-level or population effect size estimates, observed object-level effects are regularly sought to be made comparable by weighing them to the observed *s* (Schulze, 2004). But since *s* varies with the (random) influences on an empirical setting, this *invites* all the problems discussed above. So, if a meta-analysis retains the observed *s* of observed object-level effects, a robust meta-level or population effect size estimate cannot be had. For this reason, *s* should be avoided in both theory construction research and meta-analytical research.

The similarity index *I*_SIM_ fares better. First, *I*_SIM_ offers a more transparent view of observations. This can assist in improving a theoretical construct because using *I*_SIM_ and the associated 95%-SI allows distinguishing between an empirically adequate prediction (true positive; ES_THEO_ = ES_POP_) and an empirically inadequate one (false positive; ES_THEO_ ≠ ES_POP_). Making this distinction is required to decide whether a theoretical construct can be maintained, whether its theoretically predicted effect should be adjusted, or whether additional data should be collected. The last option particularly counts if available data indicate a small effect, which is generally not well-observable.

Second, the *I*_SIM_ measure and the 95%-SI help to evaluate whether a false positive prediction indicates that the population effect is under- or overestimated. After all, for all possible combinations of a theoretically predicted effect and a sample size, as long as the percentage of non-matching observations (ES_THEO_ ≠ ES_POP_) makes it unreasonable to evaluate the ES_THEO_-value as a true positive prediction, an empirically adequate prediction is more probable to fall inside the 95%-SI than not.

Third, assume that, as *n* increases, also the value of ES_OBS_ becomes increasingly more similar to the value of the true population parameter (law of large numbers). If so, then the corresponding increase in the percentage difference between a true positive and a false positive prediction goes along with an increase in the proportion of viable theoretical assumptions relative to all possible alternative theoretical assumptions. With each additional *I*_SIM_-value for a point-ES_THEO_ = *x* that falls inside the 95%-SI, therefore, it becomes more reasonable for researchers to develop a theoretical construct for *x* because “getting something right” about *x* is more probable than not.

Fourth, if additional *independent* studies happen to estimate a point-ES_OBS_ = *y* that is similar to *x*, then *I*_SIM_ continues to approximate the condition for a perfect match between prediction and observations (*I*_SIM_ = 1). The independence of additional studies entails that the approximation of *I*_SIM_ = 1 is unlikely to occur by random. Consequently, a researcher’s confidence that ES_THEO_ = *x* is empirically adequate would increase. The same rationale underlies having confidence in a meta-analytically estimated point-ES_OBS_ that is based on independently observed object-level effects (Hunter and Schmidt, 2004).

The use-value of an *I*_SIM_-based evaluation of a theoretical construct is perhaps most readily apparent in the context of the *research program strategy* (RPS) (Witte and Zenker, 2017; Krefeld-Schwalb et al., 2018). If the effects of several independent and topically related studies are observed under low error-rates, then RPS induces the observed mean effect as a parameter estimate (see the subsection *Parameter Estimation*). Next, RPS develops a theoretical construct that logically entails a theoretically predicted point-effect of identical size as this inductive parameter estimate. Provided *new* observations under low error-rates, finally, if the likelihood of the theoretically predicted effect sufficiently exceeds the likelihood of an alternative effect, then RPS evaluates the former as *preliminarily* verified, respectively as *substantially* verified if the likelihood of the theoretically predicted effect is sufficiently similar to the maximum likelihood of new observations. For the verification thresholds of this statistical likelihood model, see Krefeld-Schwalb et al. (2018, p. 22).

Beyond this likelihood model, the attempt to verify a theoretically predicted effect by comparing it to observations *requires* an *I*_SIM_-like measure. An inductive parameter estimate, after all, has uncertainty bounds that reflect the variance of observations, whereas a theoretical construct that is developed based on theoretical considerations predicts a point-specific effect. For this reason, *I*_SIM_ avoids comparing the theoretically predicted effect *indirectly* to observations, an indirectness that results from using a statistical error account and a data distribution (e.g., a *t*-, *F*-, or *Х*^2^-distribution). Instead, the theoretically predicted effect is compared *directly* to observations (as measured), while the admissible variation of a theoretical construct is captured by the 95%-SI (see the section “*Case study”*).

This explains why we modeled the admissible variation of a theoretical construct by simulating random samples of possible measurements, rather than by using an inferential statistical theory (e.g., a likelihood model). In RPS, the inferential statistical evaluation of (simulated or real) observations is useful, only if the *I*_SIM_-value already lies within the 95%-SI, indicating that the theoretically predicted effect is similar to observations. Thus, *I*_SIM_ evaluates the similarity between a theoretical construct and observations *before* inferentially testing the theoretically predicted effect (Witte and Heitkamp, 2006). Nevertheless, for a specific theoretically predicted effect to be accepted as empirically adequate, both its point-specification and its statistical substantial verification are required. In brief, *I*_SIM_ assists in specifying the effect size, while RPS verifies it.

### Limitations

Rather than replacing standardized effect size measures such as Cohen’s *d* or inductive data-evaluation tools like a model-fitting index, *I*_SIM_ complements them. *I*_SIM_ should be applied mindfully. Several limitations apply:

First, *I*_SIM_ does not offer a criterion for a data-based decision to accept or reject hypotheses. Rather than comparing two hypotheses (H_0_, H_1_) in view of data, *I*_SIM_ evaluates only the H_1_-hypothesis that states ES_THEO_. Therefore, *I*_SIM_ cannot enable a relative statistical corroboration of a theoretical construct against random influences. This continues to require statistical testing.

Second, if the theoretically predicted effect ES_THEO_ = *x* falls outside the 95%-SI, then *x* appears to be empirically *inadequate*. This appearance may mislead researchers to prematurely abandon *x* as a candidate value for ES_THEO_. But as a rule, the decision to abandon *x* should squarely depend on having collected an adequately large sample.

Third, like all formal measures, *I*_SIM_ is open to “tweaking” the data to let ES_OBS_ and ES_THEO_ match artificially. With a new formal measure, therefore, additional temptation to engage in questionable research practices may arise.

Fourth, a simple “recycling” of the ES_OBS_-value as the ES_THEO_-value would *trivially* satisfy the perfect-match condition (*I*_SIM_ = 1), known as *p*-harking. So, the same critical considerations apply as were stated immediately above (Kerr, 1998).

Fifth, in the context of a confirmatory factor analysis (CFA), which relies on an explorative factor analysis (EFA) to evaluate the deviation of predetermined parameters in some complex mathematical model, several of these parameters must be determined simultaneously (e.g., the number and correlations of factors, their weights, loadings, etc.). However, *I*_SIM_ cannot be applied to test whether the complex mathematical model itself agrees with the abstract data deduced from it; *I*_SIM_ can only test whether a basic parameter (e.g., a mean or a correlation) agrees with empirical data. Given a correlation matrix, for instance, *I*_SIM_ can evaluate the similarity between a single predicted correlation and an empirically observed correlation (see Supplementary Appendix S2, personality traits and life outcomes). As a basic (non-complex) measure, *I*_SIM_ thus operates at the level of each element in a correlation matrix and can there compare a prediction *directly* with observations (see Perez-Gil et al., 2000).

## Conclusion

The identification of an empirically adequate theoretical construct requires determining whether a theoretically predicted effect is sufficiently similar to an observed effect. To this end, we proposed *I*_SIM_ and the 95%-SI as a simple measure to evaluate the similarity between a theoretically predicted effect and observations, a measure that avoids the statistical element of the observed standard deviation. Using computer simulations, we estimated the sample size and the observed effect size that are necessary to identify an empirically adequate theoretical construct.

Generally relevant for theory construction research, the *I*_SIM_ measure and the 95%-SI particularly serve to develop a point-specific theoretical construct, where both should be applied alongside a statistical corroboration measure (e.g., the likelihood ratio). If the *I*_SIM_-value falls within the 95%-SI, then a theoretical construct postulating a theoretically predicted point-specific effect ES_THEO_ = *x* can be (fallibly) maintained as empirically adequate. If independent studies subsequently observe a point-effect ES_OBS_ = *y* that is similar to *x*, a researcher’s confidence that *x* is empirically adequate would increase. Whereas if too many *I*_SIM_-values fall outside the 95%-SI as the number of independent studies increases, then ES_THEO_ = *x* must be corrected, or the standard error must be reduced, e.g., by restricting the experimental setting. The most direct way of reducing the standard error, of course, is to increase the sample.

An exemplary application of *I*_SIM_ to recent meta-analytical findings on the precognition effect (Bem et al., 2016) indicated that 51 peer-reviewed object-level studies individually fail to provide the evidence that is required to evaluate as empirically adequate a theoretical construct that predicts a precognition effect of *d* = 0.20 (additional application examples are found in Supplementary Appendix S2).

In behavioral science as elsewhere, measurement comprises an ontological aspect related to the theoretical construct under development, and an epistemological aspect related to the specific measurement procedures employed. When using Cohen’s *d* measure, behavioral scientists tend to address a question that combines both aspects of measurement. This is understandable if theory-testing relies on statistical inference procedures, which simultaneously relate to both aspects of measurement. But to facilitate theory construction research and the development of measurement, the ontological and epistemological aspects are best kept separate. Otherwise, it is quite difficult to say what a measurement instance in fact measures.

## Data availability statement

The datasets presented in this study can be found in online repositories. The names of the repository/repositories and accession number(s) can be found at: https://osf.io/rgwsp/.

## Author contributions

EHW developed the *I*_SIM_ measure. AS coded and ran the simulations. All authors drafted the manuscript, which FZ edited. All authors approved the final submitted version.

## Funding

FZ acknowledges support from TUBITAK (No. 118C257).

## Conflict of interest

The authors declare that the research was conducted in the absence of any commercial or financial relationships that could be construed as a potential conflict of interest.

## Publisher’s note

All claims expressed in this article are solely those of the authors and do not necessarily represent those of their affiliated organizations, or those of the publisher, the editors and the reviewers. Any product that may be evaluated in this article, or claim that may be made by its manufacturer, is not guaranteed or endorsed by the publisher.

## Footnotes

^1^The creative process of developing a theoretical construct is what C.S. Peirce called an *abduction* over past observations (Peirce, 1931–1958). Jointly with initial and boundary conditions, a theoretical construct allows for a *deduction* of a theoretical prediction about possible future observations. And a (dis-)confirmation of this prediction by *new* observations that are (in-)consistent with it relies on a testing process that the late C.S. Peirce called *induction*. Abduction thus is "the process of forming an explanatory hypothesis [and is] the only logical operation which introduces any new idea," whereas "deduction merely evolves the necessary consequences of a pure hypothesis," while induction "does nothing but determine a [truth] value" (Peirce, 5.171). Importantly, the information content of a theoretical construct exceeds that of an *inductive generalization* (e.g., a mathematical function stating an observed law-like regularity) that *descriptively* subsumes past observations. Theoretical constructs acquire this excess content by featuring at least one *theoretical entity* that is not presupposed by the observational theory employed to make past observations (see Andreas, 2021, and our Supplementary Appendix S2, dissonance theory). Behavioral scientist, however, who likewise develop theoretical constructs based on past observations, regularly fail to acknowledge, and to perform, what Hempel (1988) called *theoretical (or inductive) ascent*, i.e., "[…] a transition from a data sentence expressed in [an antecedent vocabulary] *V*_A_ to a theoretical hypothesis […]" (p. 150) that is "formulated with the help of a theoretical vocabulary, *V*_C_, whose terms refer to the kinds and characteristics of the *theoretical* entities and processes in question" (p. 147, *italics added*) which themselves are the products of *abduction*. Without theoretical ascent, therefore, the information content of a *non*-genuine theoretical construct is at most as large as that of an inductive generalization. The main consequence is that the act of predicting *future* observations based on the inductive generalization that a *non*-genuine theoretical construct is, runs straight into Hume’s problem of induction (Hume, 1739): a non-pragmatic justification for a prediction of future observations based on an inductive generalization of past observations (*sans* theoretical ascent) presupposes that induction is a *valid* mode of reasoning. But this inference is circular (Henderson, 2020).

^2^With ‘*a*’ for the *origin* of the scale (normalization), ‘*u*’ for the *unit* of the scale (standardization), and ‘*x*, *y*, *z*’ for arbitrary *measurement values*, the ratio [(*x* + *a*) *u* − (*y* + *a*) *u*] / [(*z* + *a*) *u* − (*y* + *a*) *u*] = (*x* − *y*) / (*z* − *y*) is invariant for all values of *a* and *u*.

^3^Such rigor often cannot be achieved. Researchers in personality psychology, for instance, typically cannot randomly allocate study participants according to their personality characteristics. The object of inquiry, therefore, are not treatment effects but correlations between variables. These correlations can nevertheless be generalized to a population if a sample is representative of it (Kish, 1965).
