# Supplementary material for: Predicted as observed? How to identify empirically adequate theoretical constructs
Source: Front Psychol. 2022 Dec 1;13:980261. doi: 10.3389/fpsyg.2022.980261 (PMC9751867; doi:10.3389/fpsyg.2022.980261)
Supplement: Supplementary file 1 [file Data_Sheet_1.pdf]

Appendix 1: Applying I_{SIM} under different research designs

Instances of ES_{OBS} generally vary with the research design. Schäfer and Schwarz (2019, Table 3), for example, draw on a large sample of published instances of ES_{OBS} in pre-registered studies and control for the true effect size before an experimental intervention is deployed. They report a median ES_{OBS} / s of d = 0.35 for studies using a *between-subjects* design, compared to a median ES_{OBS} / s of d = 0.70 for studies using a *within-subject* design.

Here we describe the application of I_{SIM} under four different experimental (A1-A4) and one correlational design (B). As is explained in the main text, if the I_{SIM}-value falls within the 95%-SI [0.80; 1.20], then the ES_{THEO}-value can be maintained as a preliminarily empirically adequate prediction. Whereas if the I_{SIM}-value falls outside the 95%-SI, then ES_{THEO} should be adjusted or the error-rates (α, β) should be reduced by increasing the sample. (See Appendix 2 for specific applications.)

A. Experimental designs

In all four experimental designs, steps one and two are the same.

Common steps

1. Establish the experimental condition under which the empirical adequacy of a theoretical construct is to be evaluated.
2. Use past empirical results, assumptions, or intuitions, to point-specify ES_{THEO} on the measurement scale of the dependent variable.

A1. Classic control group study

3. Use a sufficiently large sample to achieve $\alpha = \beta < 0.05$, then measure the observed means m_1 and m_0 (in treatment and control group) and use the theoretically predicted m_{THEO} to calculate $I_{SIM} = (m_{THEO} – m_0) / (m_1 – m_0)$.
4. From Table 1 (see main text), extrapolate the percentage of I_{SIM}-values that fall within the 95%-SI under the assumption that ES_{THEO} predicts ES_{POP}.

A2. Between-within-subjects design, two study groups (2x2)

3. Before the experimental intervention, ensure that the pre-conditions in the treatment and the control group are sufficiently similar (e.g., the experimental condition itself, the test location, the equipment, the time of day, etc.).
4. Using a sufficiently large sample to achieve $\alpha = \beta < 0.05$ given ES_{OBS}, establish the observed pre- and post-intervention means in the treatment and the control group.
5. For the post-intervention condition, calculate $I_{SIM} = (m_{THEO\text{-}POST} – m_{0\text{-}POST}) / (m_{1\text{-}POST} – m_{0\text{-}POST})$.

A3. Between-subjects design with three or more study groups (e.g., 2 x 2 x 2; 3 x 2)

3. Ensure that the pre-conditions are similar (see A2, step 3).
4. Using a sufficiently large sample to achieve $\alpha = \beta < 0.05$, calculate the observed means for each intervention separately in the treatment and the control group.

5. Calculate I_{SIM} for each treatment group as $I_{SIM} = (m_{THEO} - m_0) / (m_1 - m_0)$.

A4. Repeated-measure design (within-subjects design)

3. See instructions for the between-subjects design (see A3, step 3).
4. Since $s^2_{diff} = s^2_1 + s^2_0 - 2\, r_{10} \times s_1 \times s_0$, if the observed correlation (r_{10}) between repeated measures is positive, then the standard deviation will be smaller than in a between-subjects design (where $r_{10} = 0$). Compared to a within-subjects design, therefore, the ES_{OBS}-value increases. Use this increased ES_{OBS}-value to extrapolate the I_{SIM} -value from Table 1 in the main text.

B. Correlational design

B1. Non-experimental study comparing two correlations

1. Use past empirical results, assumptions, or intuitions to specify the expected observed correlation given the theoretical construct, respectively without it.
2. For both cases, establish the empirical correlations. (If a zero-correlation is theoretically expected, collect only a single data set.)
3. Compute I_{SIM} as a normally distributed Fisher-*z*-transformed value, i.e., transform $-1 \leq r \leq +1$ into $-\infty \leq z \leq +\infty$.
4. In this case, the I_{SIM}-values in Table 1 (see main text) can only provide a rough approximation because a correlation is based on two columns of data-points. Compared to using a single column (as when estimating a mean), this increases the error variance because a correlation (i.e., the product of two *z*-transformed vector elements) is the mean of elements that belong to two distributions. If both columns of data points are correlated to $r \geq 0.50$, the increased error variance is compensated for. Otherwise, the ES_{OBS}-value of a true correlation may be more strongly underestimated than the ES_{OBS}-value of an observed mean that relies on only a single column of data points.
5. Provided the theoretical construct models the dependent variable on a scale of *z*-values, evaluate the similarity between ES_{THEO} and ES_{OBS} as above, by extrapolating the I_{SIM} -value from Table 1.

Appendix 2: Additional examples

1. Dissonance theory

1.1 Cohen (1962)

Independently observed empirical findings provide important test cases to construct a theory that retrodicts these findings (Witte, 1980; 1994; 1996; Witte & Heitkamp, 2006). According to a verbal version of *dissonance theory* (Festinger, 1957), for instance, experiencing discomfort motivates people to *reduce* the perceived discrepancy between their identity and a task perceived to threaten it. Using a classical forced compliance setting, Cohen (1962) offered groups of college students either a \$10, \$5, \$1, or \$0.5 reward for writing an essay in *support* of a policy that would allow the local police to keep students from protesting on campus (experimental group).[1] The control group received no reward and did not write an essay.

The relevant magnitudes for this setting are the students' *actual attitude before* writing the essay (measure A), the *forced attitude* expressed in the essay (measure B), and the *actual attitude after* writing it (measure C). On a seven-point Likert scale, a measured value > 4 thus indicates a "police-friendly" attitude. Importantly, independent attitude ratings failed to reveal significant differences between the four reward groups on the measures A and B.

Unlike *reinforcement theory* (Domjan, 2003), dissonance theory predicts that if the display of behavior *contrary* to one's actual attitude is *insufficiently* rewarded, then dissonance is reduced by shifting *away* from that attitude, i.e., towards a more "police-friendly" attitude. A *sufficient* reward, by contrast, acts like a bribe, wherefore the actual pre-task attitude can persist even if the contrary attitude is displayed. Dissonance theory particularly predicts that a *large* reward correlates with a *small* attitude shift. A reward ≥ \$1 would thus suffice to motivate maintaining the actual attitude, whereas a \$0.5 reward fails to do so. The \$1 reward group, therefore, should be *less sensitive* to the "police-friendly" attitude expressed in the essay than the \$0.5 group, and the \$0.5 group should shift *most strongly* towards it. In the control group and the \$10 and the \$5 reward groups, by contrast—where the rewards are sufficient to motivate maintaining the pre-task attitude (measure A)—an attitude shift should *not* occur. Thus, the expectation is that measure C = measure A.

According to Anderson's (1991a,b,c; 1996) *information integration theory*, the constructive task is to determine the *functional combination* of the measures A, B, and C—i.e., the function C = *f* (A, B)—that models the average impact of the attitude expressed in the essay on the pre-task attitude given a specific monetary reward (\$0.5, \$1, \$5, \$10 *vs*. \$0). According to Cohen's (1962) data, the average rating for measure C failed to differ significantly between the \$0 control group and the \$5 and \$10 reward groups ($C_{\$0} = C_{\$5} = C_{\$10}$). The sample-weighted average attitude rating across these three reward groups, therefore, provides a good enough estimate of the actual attitude towards the police in the student population ($ES_{POP} = C_{0,5,10\$}$).

---

[1] In the 1960s, the median annual US household income was around \$5.600, increasing by a factor of 16 to around \$92.800 in 2020. This admittedly crude conversion suggests that \$0.5, \$1, \$5, \$10 would, in 2020, correspond to \$8, \$16, \$80, and \$160, a far larger reward than experimental participants are typically offered today.

To model the attitude shift, previous research suggests the weights ½ for the \$0.5 reward group, ¼ for the \$1 reward group (Witte, 1979; 1980), and a zero weight for the \$5 and the \$10 reward-dollar group ($A_{\$0,\$5,\$10} = C_{\$0,\$5,\$10}$). This yields the following three constructs as specifications of C = *f* (A, B):

(i) C = A + ½ × (B – A) [\$0.5; *strongest* attitude shift]
(ii) C = A + ¼ × (B – A) [\$1; *moderate* attitude shift]
(iii) C = A + 0 × (B – A) [\$0, \$5, \$10; *no* attitude shift]

Across all groups, the average observed attitude rating of A = 2.75 and B = 5.75 failed to differ significantly (Cohen, 1962). Moreover, the average pre- and post-task ratings of students' actual attitudes (A, C) failed to differ significantly between the \$0, \$5, and the 10\$ group. An empirically adequate theoretical construct, therefore, should predict ES_{THEO} = (A – C) = 0. Moreover, since *twice* the impact on measure A is expected in the \$0.5 as in the \$1 reward-group, the construct should predict $ES_{THEO} = C_{\$0.5} = 2.75 + ½ \times (5.75 – 2.75) = 4.25$ and $C_{\$1} = 2.75 + ¼ \times (5.75 – 2.75) = 3.50$.

These predictions compare well with $ES_{OBS} = C_{\$0.5} = 4.54$ and $C_{\$1} = 3.47$. Since all three measures presuppose the same seven-point scale, one finds for the constructs (i) and (ii): $I_{SIM(\$0.5)} = |4.25 – 2.75| / |4.54 – 2.75| = 1.5 / 1.79 = 0.84$, and $I_{SIM(\$1)} = |3.50 – 2.75| / |3.47 – 2.75| = 0.75 / 0.72 = 1.04$. For the constructs (i) and (ii), the I_{SIM}-values thus fall within the 95%-SI [0.80;1.20].

Applying I_{SIM} to the construct (iii), which predicts for the \$5 and the \$10 reward group that an attitude shift does not occur ($ES_{THEO} = m_{theo} = 0$), requires the additional assumption that the actual attitude is *at least somewhat* influenced by the forced task of writing a "police-friendly" essay. This assumption entails that the original \$0 control group does as such become inadequate. After all, since the \$1 reward group already yields the *smallest* observed attitude shift, a theory can only predict a *yet smaller* attitude shift for the \$5 and the \$10 reward groups. Given that the essay's content influences the attitude, the \$1 reward group must replace the \$0 reward group as a new control group.

Since I_{SIM}'s numerator remains ($m_{THEO} – m_{OBS}$) and because m_{obs}, itself the standardization factor for m_{THEO}, is now set to the smallest observed deviation from the pre-task attitude of $C_{\$1} = 3.47$, we find $I_{SIM(\$5)} = I_{SIM(\$10)} = |2.75 – 3.47| / |2.75 – 3.47| = 1.00$. The numerator of I_{SIM} thus represents ES_{THEO}, whereas the denominator represents ES_{OBS}. The numerator and the denominator of I_{SIM} here take a negative sign because the score for the actual attitude (measure A) is smaller—thus representing a less friendly attitude towards the police—than the value of the smallest deviation from the original attitude towards the police in the \$1 reward-group. In the numerator of I_{SIM}, therefore, one would expect A = C = 2.75.

Theoretically, then, despite students being forced to express a "police-friendly" attitude, the essay writing task (resulting in the expressed attitude scored on measure B) did on average *fail* to exert even the smallest influence on students' actual attitude towards the police (measure C). The observed attitude thus remains constant, *falsifying* the initial theoretical prediction that the forced compliance task does at least somewhat shift students' actual attitude. Consequently, a *new* theoretical prediction must be formulated,

namely ‘attitude shift fails to occur because dissonance is not experienced’. This new prediction, of course, does not provide an independent evaluation of the constructs (i-iii). It nevertheless explains why, upon being exposed to the contrary attitude that students were forced to express, their actual attitude failed to shift.

The above differs from a mere induction of observed empirical findings, i.e., from an *inductive generalization*, because the weights (½, ¼, 0) of the three focal parameters (measures A, B, C) are *not* fitted to data. Moreover, since the experimental conditions are qualitatively different, a reduction of dissonance, and hence an attitude shift, is theoretically expected to occur only under specific reward conditions. Under other reward conditions, one would expect different empirical findings. All along, one maintains a *single* theoretical model—C = *f* (A, B)—that is specified as the constructs (i-iii).

Of course, constructing this theoretical model requires extant empirical data as the relevant material to which the model can be compared. But since different empirical findings can be subsumed under a single theoretical model that postulates a set of non-fitted theoretical parameters, the model reaches *beyond* the available empirical data in the sense of our footnote 1 (see main text). After all, although measures A, B, and C are presupposed by a relevant observational theory, the specific weights and the additive function are exclusively presupposed by the theoretical model.

The same theoretical model is applied in the next subsection.

1.2 Festinger & Carlsmith (1959)

According to a second classic experimental study (Festinger & Carlsmith, 1959), students who were separated into a \$20 and a \$1 reward-group had to perform the dull task of spending 30 minutes filling and emptying trays of spools, followed by 30 minutes spent rotating pegs. Both reward groups were then forced to *mislead* other students that this dull task was interesting, enjoyable, and of scientific value. Persuasive success was measured by the other students’ displayed motivation to sign-up for a similar task.

The three scales for the task’s *dullness* are as above. Also as above, if \$1 is an *insufficient* reward, then an attitude shift is predicted to occur in the \$1 reward group, whereas it is *not* predicted to occur in the \$20 reward group—as confirmed by the observed deviation from the \$0 control group being statistically insignificant. The functional elements thus are the average ES_{OBS} for students’ actual attitude towards the task in the control group, where no persuasive attempt occurred (measure A), and the maximum of the Likert scale (measure B).[2] As before, the weights are ¼ for the \$1 reward group and zero for the \$20 reward group.

---

[2] A scale’s width and its endpoints invariably provide participants with an orientation as to the meaning of a scale’s numerical values (Anderson, 1996). Of course, a functional specification of a theoretical construct is meaningful, only if the numerical values that are measured on *different* scales are standardized to a scale’s endpoint, as has here occurred for the three sub-scales measuring the *dullness* of the task (measure B). The sub-scales are *interesting task* (width −5 to +5; in the control and the \$20-group, the means are A = –0.25, the endpoint is B = +5), *scientific importance* (0 to 10, A = 5.39,

When these elements are combined into a point-specific theoretical model, then the ES_{THEO} on all three scales is:

(iv) C = A + ¼ × (B – A)

As to the task's *dullness*, model (iv) predicts C = –0.25 + ¼ (5.00 + 0.25) = 1.06. Compared to ES_{OBS} = 1.35, we find I_{SIM} = |1.06 + 0.25| / |1.35 + 0.25| = 0.82. As to the task's *scientific value*, model (iv) predicts C = 5.39 + ¼ (10 – 5.39) = 6.54. Compared to ES_{OBS} = 6.45, we find I_{SIM} = |6.54 – 5.39| / |6.45 – 5.39| = 1.08. As to being *motivated* to sign-up for a similar task, model (iv) predicts C = –0.44 + ¼ (5.0 + 0.44) = 0.92. Compared to ES_{OBS} = 1.20, we find I_{SIM} = |0.92 + 0.44| / |1.20 + 0.44| = 0.83. Thus, I_{SIM} shows that model (iv) both *under*- and *over*estimates ES_{OBS}. Given that the deviations from I_{SIM} = 1 are rather small, however, ES_{THEO} and ES_{OBS} are similar enough to consider model (iv) empirically adequate.

This demonstrates how, given different conditions and studies, I_{SIM} can evaluate a classical theory by applying it to a fixed theoretical model.

2. Crowdsourcing hypothesis tests

If the scientific gold standard is the successful replication of an original ES_{OBS} under *different* empirical conditions, then independent labs must somehow converge upon the ES-value that an empirically adequate ES_{THEO} would predict. To this end, "[c]rowdsourcing study designs using many research teams can help reveal the conceptual robustness of the effects" (Landy et al., 2020, 3). In Landy et al.'s (2020) own design, up to thirteen labs—each using samples of around n = 300 per group— attempted to replicate the original ES_{OBS} for the following (directionally unspecified) hypotheses or research questions (H_1-H_4) (Landy et al., 2020, 6):

- H_1: When asked directly, do people explicitly self-report awareness of harboring negative automatic associations for members of negatively stereotyped social groups?
- H_2: Compared to a negotiator who makes a moderate first offer, is a negotiator who makes an extreme first offer trusted more, less, or the same?
- H_3: How are an individual's moral judgments affected by continuing to work despite lack of material/financial need—beneficial, detrimental, or no effect?
- H_4: Part of the reason for opposing the use of performance-enhancing drugs in sports is that this is "against the rules." But which factor contributes more to this judgment—that using such drugs is against the law or that it is against the rules established by a more proximal authority (e.g., the league)?

In all labs, the eight original and replication studies addressing H_1-H_4 each resulted in statistically significant ($p < 0.001$), but prima facie very heterogeneous observed

endpoint B = 10), *motivated to sign-up for a similar experiment* (–5 to +5, A = –0.44, endpoint B = +5).

original effects (ES_{ORI}), as well as comparably heterogeneous *replication* effects (ES_{REP}). Since $ES_{OBS} = (m_1 - m_0)$, failure to observe a *robust* ES implies a large variation of the mean difference or of the standard deviation (s). To evaluate the empirical adequacy of ES_{THEO}, of course, the crucial magnitude is ($m_{THEO} - m_0$), because s merely serves to interpret ES_{OBS} relative to a statistical error theory.

Given a measurement error of $\alpha = \beta = 0.05$, a statistical evaluation of the ES_{OBS}-instances reported in Landy et al. (2020) requires comparing these instances to some (conventionally determined) *minimum* value of $ES_{THEO} / s = d_{THEO\text{-}MIN}$. This value serves to develop an empirically adequate theoretical construct given error-prone data. (Recall that the d-measure reports a statistic that combines the effect size with the measurement error s (see main text).) As a statistic, a value of $d_{THEO\text{-}MIN} = 0.80$, or what Cohen (1977) calls a *large* effect, suffices to explain around 14% of the observed variance given the influence of errors on observations. Therefore, $d_{THEO\text{-}MIN} = 0.80$ can be considered to state a mean difference *just large enough* to be promising as an empirically adequate construct.

To eliminate the variation of s across labs, all reported d-values can be averaged such that $s' = \sum s_i / N$ (with 's'' for the average). This allows computing $ES_{THEO\text{-}MIN}$ as $d_{THEO\text{-}MIN} = 0.80 \times s'$ for each of Landy et al.'s (2020) four original studies.[3] Under the assumption that $ES_{THEO} = ES_{POP}$, one can now set

$$I_{SIM} = ES_{THEO\text{-}MIN} / ES_{OBS} = [(m_{THEO} - m_0) / s'] / [(m_1 - m_0) / s'].$$

As before, ES_{OBS} is sufficiently similar to $ES_{THEO\text{-}MIN}$ if the I_{SIM}-value falls within the 95%-SI [0.80; 1.2]. Each of Landy et al.'s (2020) labs having used samples of around $n = 300$, if $ES_{THEO\text{-}MIN} = (d_{THEO\text{-}MIN} \times s') = 0.80 \times s'$ were identical in size to ES_{POP}, then almost 99% of I_{SIM}-values would fall within the 95%-SI (see Table 1, main text). Once H_1-H_4 are point-specified, however, *none* of Landy et al.'s (2020) findings suggest a promising ES_{THEO} for H_4. In the case of H_1-H_3, moreover, *far too few* findings suggest that $ES_{THEO} = d_{THEO} = 0.80$ promises to be empirically adequate by potentially matching $ES_{OBS} = d_{OBS} = 0.80$.

In the case of H_1 ("awareness of automatic prejudice"), setting $ES_{ORI} = ES_{THEO}$ and $ES_{REP} = ES_{OBS}$ results in thirteen instances where $I_{SIM} = 0$ (negative differences in the denominator of I_{SIM}), eleven instances where $I_{SIM} < 0.80$, and two instances where the I_{SIM}-value falls inside the 95%-SI ($I_{SIM} = 1.10$; 0.84; both from the same lab).

In the case of H_2 ("extreme first offers reduce trust"), ES_{ORI} and ES_{REP} result in zero instances where $I_{SIM} = 0$, in one undefined result (no difference in the denominator of I_{SIM}), in 19 instances where the I_{SIM}-value falls outside, and four instances where it falls inside the 95%-SI ($I_{SIM} = 0.84$; 1.15).

In the case of H_3 ("moral praise for needless work"), ES_{ORI} and ES_{REP} result in three instances where $I_{SIM} = 0$ (negative differences in the denominator of I_{SIM}), one undefined result (same means in the denominator of I_{SIM}), 18 instances where I_{SIM}-values

---

[3] $ES_{THEO\text{-}MIN} = d_{THEO} = 0.80$ because $d_{THEO} = (m_1 - m_2) / s$, where $s' = 1$, as all d-values are z-transformed measurements. Thus, the ES is identical to d. Since ($m_1 - m_2$) depends on n, Table 1 (main text) differentiates between measurements based on n.

falls outside the 95%-SI, and four instances falling inside (I_{SIM} 1.17; 1.20; all from the same lab).

In the case of H_4 ("proximal authorities drive the legitimacy of performance-enhancing drugs"), ES_{ORI} and ES_{REP} result in seven instances where the I_{SIM}-values are zero. The remaining seventeen I_{SIM}-values fall outside the 95%-SI.

Using a constant theoretical parameter that is derived from individual empirical results thus indicates that the data are highly heterogeneous. Consequently, one cannot reasonably proceed to develop a theoretical model.

3. Personality traits and life outcomes

A major topic in psychological research is the correlation (r) between the Big Five personality traits (*extraversion*, *agreeableness*, *conscientiousness*, *neuroticism*, *open-mindedness*) and various life outcomes (e.g., *subjective well-being*, *family satisfaction*, *occupational interests*, *leadership*, etc.). Often cited in evidence-based policymaking, this correlation has been addressed in only a single comprehensive meta-analytical replication study (Soto, 2019). This study coordinated several labs that independently reported the ES_{REP} observed in two replication studies ($ES_{REP-1,2}$) for 78 instances of an original ES_{OBS} (ES_{ORI}). After eliminating two correlational studies because of their very small samples, we retain 152 pairwise comparisons of ES_{ORI} with ES_{REP-1} and ES_{REP-2} ($2 \times 78 - 2 \times 2 = 152$).

In analogy to I_{SIM}, one can relate ES_{ORI} to ES_{REP} as the ratio $I_{SIM} = ES_{REP} / ES_{ORI}$.[4] By setting $ES_{ORI} = ES_{REP}$, this ratio becomes consistent with the 95%-SI [0.80; 1.20] and thus compares a "theorized" ES_{REP} with ES_{ORI}. Provided n is sufficiently large, therefore, I_{SIM} reports precisely what is required to evaluate the empirical adequacy of a *specific* correlation between personality traits and life outcomes.

The evaluative result comprises six instances where $I_{SIM} = 0$ (i.e., the signs in the numerator and denominator of I_{SIM} differ), 90 instances where I_{SIM}-values fall outside the 95%-SI, and 56 instances where I_{SIM}-values fall inside the 95%-SI.[5] Thus, some 37% of

[4] Since the r-measure states the mean of the product of z-scores (Cohen & Cohen, 1975), the numerator and the denominator of ES_{REP} / ES_{ORI} both report the mean difference as measured on a z-scale. Soto (2019) in fact uses a rudimentary version of this ratio (see Soto 2019, Table 1).

[5] The I_{SIM}-version ES_{REP} / ES_{ORI} differs from the I_{SIM}-version where the numerator states an ES_{THEO}-instance without statistical fluctuations, whereas the denominator is subject to error-fluctuation. A replication study is subject to a *regression effect*, the size of which correlates inversely with n (Fiedler & Prager, 2018). This effect entails that the numerator of ES_{REP} is smaller than that of ES_{ORI}. To account for the regression effect, therefore, the lower bound of the 95%-SI [0.80;1.20] should be adjusted to a *more lenient* value. In Soto's (2019) data, I_{SIM}-values < 0.80 are obtained in 60 out of 90 cases. Some 70% of ES_{REP}-values thus are smaller than the corresponding ES_{ORI}-instance. The Spearman-rank correlation between ES_{ORI}- and ES_{REP}-instances is ρ=0.34. Hence, there is a match between ES_{ORI} and ES_{REP}, but a reduction of the amount. Given the regression effect, then, the number of studies where I_{SIM}-values fall inside a *more lenient* SI-

ES_{REP}-instances are similar enough to ES_{ORI}, among which only a single study lacks a sufficiently large sample size ($n_{ORI} = 164$, $n_{REP} = 181$; $ES_{REP} = r = 0.20 = d = 0.40$). In all other cases, especially the replication samples are by far large enough (n = 1550, 1549, 1514, 1505, 1503, 944, 747, 512).

References

Anderson, N.H. (1991a). *Contributions to information integration theory*. Vol. I: *Cognition*. Hillsdale: LEA.

Anderson, N.H. (1991b). *Contributions to information integration theory*. Vol. II: *Social.* Hillsdale: LEA.

Anderson, N.H. (1991c). *Contributions to information integration theory*. Vol. III: *Developmental*. Hillsdale: LEA.

Anderson, N. H. (1996). *A functional theory of cognition*. Mahwah: LEA.

Cohen, A.R. (1962). An experiment on small reward for discrepant compliance and attitude change. In: J. W. Brehm, & A.R. Cohen (Eds.). *Exploration in cognitive dissonance* (pp. 73–78). New York: Wiley.

Cohen, J. (1977, rev. ed., 1988). *Statistical power analysis for the behavioral sciences*. London: Academic Press.

Domjan, M. (2003). *The principles of learning and behavior* (5th ed.). Belmont, CA: Thomson/Wadsworth.

Festinger, L. (1957). *A Theory of Cognitive Dissonance*. Evanston: Stanford University Press.

Festinger, L., & Carlsmith, J. M. (1959). Cognitive consequences of forced compliance. *Journal of Abnormal and Social Psychology, 58,* 203–210. https://doi.org/10.1037/h0041593

Fiedler, K., & Prager, J. (2018). The regression trap and other pitfalls of replication science—illustrated by the report of the Open Science Collaboration. *Basic and Applied Social Psychology, 40*(3), 115–124. https://doi.org/10.1080/01973533.2017.1421953

Landy, J. F., Jia, M. (L.), Ding, I. L., Viganola, D., Tierney, W., Dreber, A., Johannesson, M., Pfeiffer, T., Ebersole, C. R., Gronau, Q. F., Ly, A., van den Bergh, D., Marsman, M., Derks, K., Wagenmakers, E.-J., Proctor, A., Bartels, D. M., Bauman, C. W., Brady, W. J., . . . Uhlmann, E. L. (2020). Crowdsourcing hypothesis tests: Making transparent how design choices shape research results. *Psychological Bulletin*, 146(5), 451–479. https://doi.org/10.1037/bul0000220

Schäfer, Th., & Schwarz, M.A. (2019). The meaningfulness of effect sizes in psychological research: Differences between sub-disciplines and the impact of potential biases. *Frontiers in Psychology, 10,* 813. https://doi.org/10.3389/fpsyg.2019.00813

Soto, C. J. (2021). Do Links Between Personality and Life Outcomes Generalize? Testing the Robustness of Trait–Outcome Associations Across Gender, Age, Ethnicity, and Analytic Approaches. *Social Psychological and Personality Science, 12*(1), 118–130. https://doi.org/10.1177/1948550619900572

---

interval may suffice to evaluate for each individual study whether ES_{REP} lets ES_{ORI} appear as an empirical adequate ES_{THEO}.

Witte, E.H. (1979). *Das Verhalten in Gruppensituationen. Ein theoretisches Konzept*. Göttingen: Hogrefe.

Witte, E.H. (1980). Die Theorie der kognitiven Dissonanz und das Konzept der Informationsintegration: Zur Entwicklung einer gemeinsamen Theorie. In: E.H. Witte (Ed.), *Beiträge zur Sozialpsychologie* (pp. 233–251). Weinheim: Beltz.

Witte, E.H. (1994). Minority influences and innovations: The search for integrated explanation of psychological and sociological models. In: S. Moscovici., A. Mucchi-Faina, & A. Maass (eds.), *Minority influence* (pp. 67–93). Chicago: Nelson-Hall.

Witte, E.H. (1996). The extended group situation theory (EGST): Explaining the amount of change. In: E.H. Witte, & J.H. Davis (eds.). *Understanding group behavior* (Vol. 1) (pp. 253–291). Mahwah: Erlbaum.

Witte, E.H., & Heitkamp, I. (2006). Quantitative Rekonstruktionen (Retrognosen) als Instrument der Theorienbildung und Theorienprüfung in der Sozialpsychologie. *Zeitschrift für Sozialpsychologie*, *37*(3), 205–214. https://doi.org/10.1024/0044-3514.37.3.205
